# CKD-5, a novel pan-histone deacetylase inhibitor, synergistically enhances the efficacy of sorafenib for hepatocellular carcinoma

**DOI:** 10.1186/s12885-020-07471-3

**Published:** 2020-10-15

**Authors:** Young Chang, Yun Bin Lee, Eun Ju Cho, Jeong-Hoon Lee, Su Jong Yu, Yoon Jun Kim, Jung-Hwan Yoon

**Affiliations:** 1 Department of Internal Medicine and Liver Research Institute, Seoul National University College of Medicine, 101 Daehak-ro, Jongno-gu, Seoul, 03080 Republic of Korea; 2 Institute for Digestive Research, Digestive Disease Center, Department of Internal Medicine, Soonchunhyang University College of Medicine, Seoul, Korea

## Abstract

**Background:**

Histone deacetylase inhibitors (HDACIs) have distinctive epigenetic targets involved in hepatocarcinogenesis and chemoresistance. A recent phase I/II study reported the possibility of HDACI as a chemosensitizer in sorafenib-resistant patients. In this study, we evaluated whether CKD-5, a novel pan-HDACI, can potentiate the efficacy of sorafenib.

**Methods:**

The anticancer effect of CKD-5 with and without sorafenib was evaluated in vitro using an MTS assay with human HCC cells (SNU-3058 and SNU-761) under both normoxic and hypoxic conditions. Microarray analysis was performed to investigate the mechanism of cell death, which was also evaluated by small interfering RNA (siRNA) transfection and subsequent immunoblot assays. In vivo experiments were conducted using two different murine HCC models. C3H mice implanted with MH134 cells and C57BL/6 mice implanted with RIL-175 cells were treated with weekly CKD-5 with and without sorafenib for 2 weeks.

**Results:**

CKD-5 treatment significantly suppressed human HCC cell growth in both normoxic and hypoxic conditions. Microarray analysis and real-time PCR showed that CKD-5 treatment significantly increased peripherin expression in HCC cells and that downregulation of peripherin by siRNA decreased CKD-5-induced apoptosis. The combination of CKD-5 and sorafenib decreased cell viability more effectively than sorafenib or CKD-5 monotherapy in human and murine HCC cells. The effectiveness of the combination therapy was consistently demonstrated in the animal models. Histological and biochemical analyses demonstrated good tolerance of CKD-5 plus sorafenib in vivo.

**Conclusion:**

CKD-5 may enhance sorafenib efficacy through epigenetic regulation. The combination of CKD-5 and sorafenib might be a novel therapeutic option for the treatment of HCC.

## Background

Hepatocellular carcinoma (HCC) is the most common primary malignancy of the liver. Liver cancer is predicted to be the fifth most commonly diagnosed cancer and the second leading cause of cancer mortality worldwide in 2018 [1]. Owing to its global disease burden and poor prognosis, HCC remains a major global health problem. There are various effective treatments for early to intermediate stage HCC such as surgical resection, radiofrequency ablation, trans-arterial chemoembolization, and transplantation. In contrast, patients with advanced HCC have very poor prognoses and limited effective treatments. Sorafenib, a multi-kinase inhibitor, was established as a standard treatment for advanced HCC patients based on the results of the SHARP (Sorafenib HCC Assessment Randomized Protocol) trials [2]. In clinical practice, however, there have been limitations in treating advanced HCC patients with sorafenib because it has demonstrated limited survival benefits with low tumor response rates, suggesting the existence of sorafenib-resistance [3]. To overcome these limitations of sorafenib, combination therapies with various treatment modalities such as locoregional treatment [4–6], conventional chemotherapy [7–9], and novel targeted agents [10–12] have been tried.

Histone deacetylase inhibitors (HDACIs) have been extensively studied and are considered promising anticancer agents based on preclinical and clinical trials [13, 14]. Many clinical trials have proved the efficacy of HDACIs in hematologic malignancies, including various kinds of lymphoma, and in thymoma and melanoma [14]. Based on the positive results of these clinical trials, the US FDA firstly approved panobinostat, one of HDACIs, for treatment of progressive or relapsed T cell lymphoma in the United States in 2006; it was approved in Korea in 2012. Also, HDACI efficacy against advanced HCC has been suggested in a preclinical study [15].

CKD-5 is a novel hydroxamic acid-based pan-HDACI under investigation for the treatment of various tumor types. In preclinical in vitro and in vivo studies, CKD-5 has demonstrated strong anti-tumor effects against multiple myeloma and cutaneous T-cell lymphoma [16, 17]. CKD-5 has also shown considerable cytotoxicity in multiple solid tumor cell lines and xenograft mouse models of colon, prostate, and lung cancer [18]. A phase I study of CKD-5 recently reported promising results with minimal side effects and modest anti-tumor efficacy in patients with lymphoma or multiple myeloma refractory to standard therapy [19]. In this study, we demonstrated that CKD-5 has consistent anti-tumor effects in various HCC cell lines and xenograft mice models, which is further potentiated when combined with sorafenib. This preclinical study establishes a rationale for clinical studies of HDACI therapy for HCC.

## Methods

### Cell lines and cell culture

Five HCC cell lines were used: Huh-7, a well differentiated HCC cell line [20]; SNU-761, a poorly differentiated HCC cell line [21]; SNU-3058, a hypovascular HCC cell line provided by the Korea Cell Line Bank; MH-134 [22] and RIL-175 (provided by Professor T. Greten, NIH) murine HCC cell lines. Cells were grown in RPMI 1640 supplemented with 10% fetal bovine serum (FBS), 100,000 U/L penicillin and 100 mg/L streptomycin with or without 100 mM insulin [23]. In all experiments, cells were serum-starved overnight before the experiments to avoid the effect of serum-induced signaling. Cells were incubated under either standard normoxic (20% O_2_ and 5% CO_2_ at 37 °C) or hypoxic (1% O_2_, 5% CO_2_, and 94% N_2_ at 37 °C) conditions. As hypoxia induced by the rapid-growing nature of HCC, plays a critical role in HCC malignance and treatment failure, we performed the experiments in both normoxic and hypoxic conditions [24].

### Reagents and animals

CKD-5, a novel HDACI, was provided by Chong Kun Dang Pharmaceutical Corp. (Seoul, Korea). Panobinostat was purchased from Novartis Pharmaceuticals (East Hanover, NJ, USA). Sorafenib was purchased from LC Laboratories (Woburn, MA, USA). C3H mice and C57BL/6 mice were purchased from Orient Bio Inc. (Seongnam, Korea) for in vivo experiments.

### Cell proliferation analysis (MTS assay)

Cell proliferation was measured on the basis of cellular conversion of the colorimetric reagent 3,4-(5-dimethylthiazol-2-yl)-5-(3-carboxymethoxyphenyl)-2-(4-sulfo-phenyl)-2H-tetrazolium salt (MTS) into soluble formazan by dehydrogenase enzyme found in metabolically proliferating cells with the Cell Titer 96 Aqueous One Solution cell proliferation assay (Promega, Madison, WI) [25]. Following each treatment, 20 μL of dye solution was added into each well in 96-well plate and incubated for 2 h. The absorbance was recorded at a wavelength of 490 nm using an enzyme-linked immunosorbent assay plate reader (Molecular Devices, Sunnyvale, CA, USA).

### Complementary deoxyribonucleic acid (cDNA) microarray analysis

To compare relative gene expression profiles in human HCC cells after HDACI treatment, total ribonucleic acids (RNAs) from Huh-7 cells treated with HDACI (M171) or control reagent were extracted and purified. Microarray analysis was performed according to the Macrogen Rat Bead Chip technical manual (Macrogen, Seoul, Korea) using an Illumina RatRef-12 Expression Bead Chip (Illumina, Inc., San Diego, CA, USA) [25]. Biotinylated cRNAs were prepared from 0.55 μg quantities of total RNA using the Illumina Total Prep RNA Amplification Kit (Ambion, Austin, TX, USA). After fragmentation, cRNAs were hybridized to the Illumina RatRef-12 Expression Bead Chip in 0.75 μg quantities using protocols provided by the manufacturer. Arrays were scanned using the Illumina Bead Array Reader Confocal Scanner. Array data export processing and analysis were performed using Illumina Bead Studio v3.1.3 (Gene Expression Module v3.3.8).

### Real-time polymerase chain reaction (PCR) analysis

Total RNA was extracted using Trizol Reagent (Invitrogen, Carlsbad, CA, USA). cDNA templates were synthesized using oligo-dT random primers and Moloney murine leukemia virus reverse transcriptase. After the reverse transcription reaction, the cDNA template was amplified by PCR using Taq polymerase (Invitrogen) [26]. Peripherin and glutathione peroxidase 4 (GPX4) were quantitated by real-time PCR (LightCycler; Roche Molecular Biochemicals, Mannheim, Germany) using SYBR green as the fluorophore (Molecular Probes, Eugene, OR, USA). After electrophoresis in 1% agarose gel, the portion of gel containing the expected peripherin PCR product was excised, and the product was eluted into Tris-HCl using a DNA elution kit (Qiagen, Valencia, CA, USA). Primers for peripherin were AGCTACTGGAAGGGGAGGAG (forward) and CGGGTCTCAATTGTCCTGAT (reverse) [27, 28], and primers for GPX4 were TAAGAACGGCTGCGTGGTGAAG (forward) and AGAGATAGCACGGCAGGTCCTT (reverse) [29]. Glyceraldehyde-3-phosphate dehydrogenase (GAPDH) gene expression was used as a control. Peripherin mRNA expression levels were calculated as the relative intensity of the PCR product bands compared with those of the GAPDH gene using the 2^–∆∆Ct^ method. All PCR experiments were performed in triplicate.

### Small interfering RNA (siRNA) transfection

Cells were seeded in a 6-well culture plate (2 × 10^5^ cells/well) in 2 mL antibiotic-free medium supplemented with 10% FBS. At 60–80% confluence, the cells were transfected with siRNA using the siRNA Transfection Reagent (Santa Cruz Biotechnology Inc., Santa Cruz, CA, USA) according to the manufacturer’s instructions. The cells were treated with siRNA for 6 h at 37 °C and growth medium containing 20% FBS and antibiotics was added. After 18 h, the medium was replaced with fresh medium containing 10% FBS and antibiotics, and 24 h after transfection, the cells were used in the subsequent experiments [25].

### Immunoblot analysis

For the immunoblot analysis, 20 nM CKD-5 was treated 24 h before, followed by treatment with 2 μM sorafenib. Cells were lysed on ice for 20 min using lysis buffer (50 mM Tris-HCl, pH 7.4; 1% Nonidet P-40, 0.25% sodium deoxycholate; 150 mM NaCl; 1 mmol/L EDTA; 1 mM phenylmethylsulfonyl-fluoride; 1 mM Na3VO4; 1 mM NaF; and 1 mg/mL each of aprotinin, pepstatin, and leupeptin) and centrifuged at 14,000×*g* for 10 min at 4 °C. 50 μg and 30 μg of protein from SNU761 and SNU3058 cells, respectively, were loaded on a 12.5% SDS-PAGE gel. Samples were transferred to nitrocellulose membranes, blotted with appropriate primary antibodies at a dilution of 1:1000, and treated with peroxidase-conjugated secondary antibodies (Biosource International, Camarillo, CA, USA). Bound antibodies were visualized using chemiluminescent substrate (ECL; Amersham, Arlington Heights, IL, USA) and exposed to Kodak X-OMAT film (Kodak, New Haven, CT, USA) [25]. Primary antibodies included rabbit anti-caspase 9 and 7 (cleaved) (Cell Signaling Technology, Danvers, MA, USA), anti-heat shock protein 90 (HSP90), anti-P21 (both from Santa Cruz Biotechnology Inc., Santa Cruz, CA, USA), anti-GPX4, and anti-acetylated H3 (both from Abcam, Cambridge, UK). A goat anti-actin antibody was also used (Santa Cruz Biotechnology Inc., Santa Cruz, CA, USA).

### In vivo subcutaneous xenograft mouse models

MH-134 cells (2.5 × 10^6^/mL in 100 μL of RPMI-1640) were injected subcutaneously into the flank of 6-week-old C3H mice (*n* = 64) [30]. Randomization into 8 groups with 8 mice each group was performed when the implanted MH-134 tumor bud reached a volume of 0.2 cm^3^ in more than 60% of mice: control, low dose of CKD-5 (40 mg/kg), high dose of CKD-5 (60 mg/kg), panobinostat (10 mg/kg), sorafenib (30 mg/kg), panobinostat (10 mg/kg) + sorafenib (30 mg/kg), low dose of CKD-5 (40 mg/kg) + sorafenib (30 mg/kg), high dose of CKD-5 (60 mg/kg) + sorafenib (30 mg/kg). CKD-5 was injected intraperitoneally once a week (D0, D7) using a solution of 0.9% saline. Panobinostat was injected intraperitoneally 3 times a week (D0, D2, D4, D7, D9, and D11) using a solution of 0.9% saline with 10% ethanol and 10% Cremophor (Sigma, St. Louis, MO) as vehicle. Sorafenib was administered per gavage once daily (D0 to D13) using 0.5% carboxymethylcellulose sodium as vehicle. Animals were euthanized on day 14 to acquire tumor, liver, spleen, and blood samples for analysis. Euthanasia was performed by introducing 100% carbon dioxide gas in the chamber with a fill rate of 10 to 30% of the chamber volume per minute.

Repeated experiments were performed using the same mouse model with increased numbers of individuals in each group. A total of 35 C3H mice implanted with MH-134 HCC cells were assigned to 4 groups: control, CKD-5 (60 mg/kg), sorafenib (30 mg/kg), and CKD-5 (60 mg/kg) plus sorafenib (30 mg/kg). After randomization into 4 groups, experiments were conducted using the same protocol as in the previous experiments. To validate the results obtained with the MH-134/C3H mouse model, another xenograft mouse model was established. RIL-175 cells (2.5 × 10^6^/mL in 100 μL of RPMI-1640) were injected into C57BL/6 mice [31], and experiments using the same protocol were performed.

Tumor volumes were measured using a Vernier caliper and calculated as a standard formula: *π* /6 x (length) x (width) ^2^ [32]. Pathologic analysis was performed using an Aperio image analyzer (Aperio Technologies, Inc., Vista, CA, USA). Staining positivity was defined as strong positive and positive results on Aperio image analysis.

### Statistical analysis

All the experimental results were obtained from at least 3 independent in vitro experiments and at least 8 mice in vivo experiments and presented as the mean ± standard deviation. For comparisons between groups, data were analyzed by student *t*-test, Mann-Whitney *U* test or one-way ANOVA. Before conducting Student’s t-test, the normal distribution of variables was verified using Shapiro-Wilk test; Levene’s test was performed to assess the homogeneity of variables between groups. Bliss independent analysis was conducted to confirm the synergistic anticancer efficacy of combination treatment [33]. For all tests, *P* < 0.05 was regarded as statistically significant. Statistical analyses were performed using PASW version 23.0 (IMB, Chicago, IL, USA).

### Ethics statement

We carried out this study in strict accordance with the recommendations in the Guide for the Care and Use of Laboratory Animals of the National Institutes of Health. The in vivo study protocol was approved by the Institutional Animal Care and Use Committee (IACUC No. 18–0077-S1A0, 17–0060-C1A0) of Seoul National University Hospital.

## Results

### CKD-5 affected cell viability and apoptosis in HCC cell lines

**Fig. 1:**
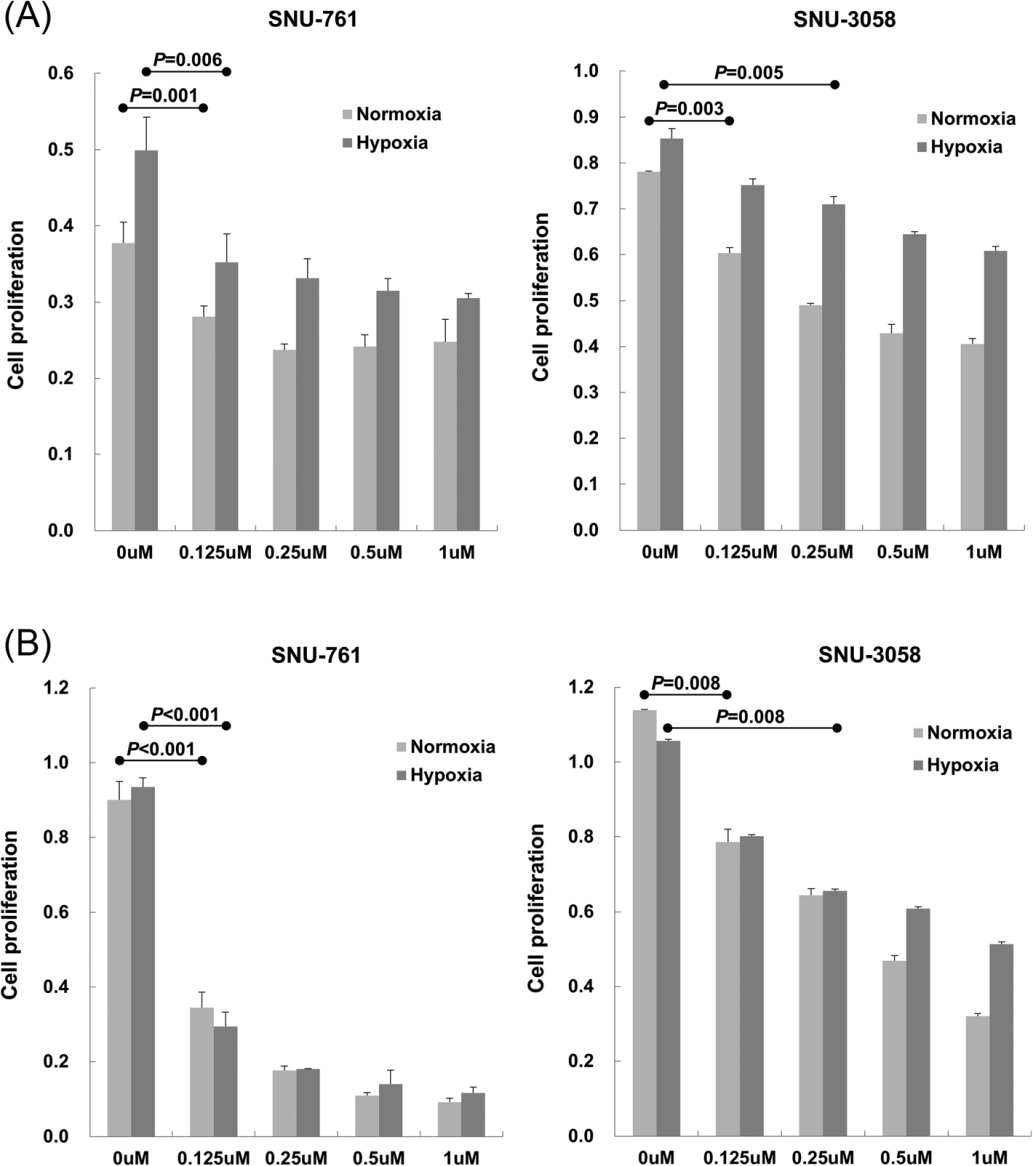
Anti-tumor effects of CKD-5 on SNU-761 and SNU-3085 after **a** 24-h and **b** 48-h incubation. CKD-5 treatment significantly attenuated HCC cell proliferation in both normoxic and hypoxic conditions in a dose-dependent manner

**Fig. 2:**
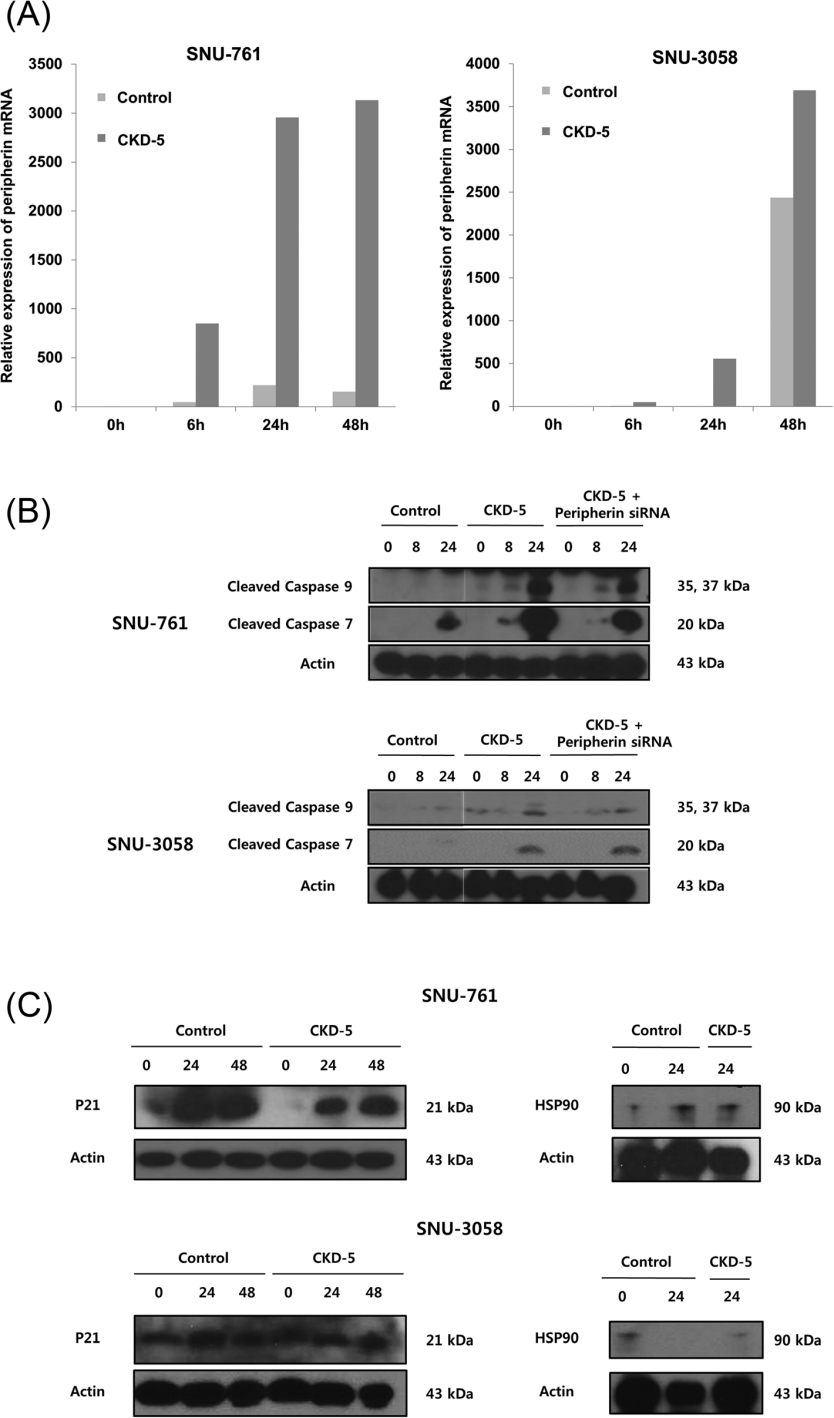
The mechanism of anti-tumor effects of CKD-5. **a** Peripherin mRNA was significantly overexpressed after CKD-5 treatment compared to control treatment in both SNU-761 and SNU-3058 cells. **b** CKD-5 treatment increased cleaved caspase-7 and -9 expression, which was downregulated after knockdown of peripherin with peripherin-specific siRNA in both SNU-761 and SNU-3058 cells. **c** Neither acetylated-HSP90 nor p21 expression was altered after CKD-5 treatment in both SNU-761 and SNU-3058 cells

CKD-5 treatment decreased SNU-761 and SNU-3085 cell proliferation in both normoxic and hypoxic conditions in a dose-dependent manner after 24-h incubation (Fig. 1a), and the anti-tumor effect was further enhanced after 48-h incubation (Fig. 1b). Also, CKD-5 enhanced apoptosis of HCC cells compared to controls, represented by increased expression of caspase-7 and -9 cleavages (Fig. 2b). The cytotoxic efficacy of CKD-5 was further compared with that of panobinostat, a previously approved HDAC inhibitor, using SNU761 cells. Both panobinostat and CKD-5 treatments reduced cell proliferation, and the cytotoxic efficacy was more potent when treated with CKD-5 than with panobinostat, especially in combination with sorafenib (Supplementary Figure 1).

### CKD-5 overexpressed peripherin in HCC cells, mediating CKD-5-induced HCC apoptosis

We sought to identify molecules that had altered expression after HDACI treatment of HCC cells to explain the mechanism of the CKD-5 anti-tumor effect. cDNA microarray analysis (Supplementary Table 1 and Supplementary Figure 2) showed significant peripherin overexpression after HDACI treatment compared to levels in controls with a 14.13-fold change. When SNU-761 and SNU-3058 cells were exposed to CKD-5, peripherin mRNA expression was significantly increased in both cell lines (Fig. 2a).

To demonstrate the effect of CKD-5-induced peripherin overexpression in HCC cells, we transfected SNU-761 and SNU-3058 cells with control or peripherin siRNA and cultured the cells with CKD-5 for 24 h. CKD-5-induced cleaved caspase-7 and -9 expression was downregulated by peripherin knockdown with siRNA (Fig. 2b), indicating that CKD-5 induces HCC cell apoptosis via peripherin overexpression. We examined the expression of P21 and acetylated-HSP90, known to be involved in anti-tumor mechanisms of HDACI, by western blot after CKD-5 treatment. Neither acetylation of HSP90 nor p21 expression was altered after CKD-5 treatment (Fig. 2c), suggesting that HSP90 and p21 are not involved in the anti-tumor mechanism of CKD-5.

### CKD-5 and sorafenib synergistically inhibited HCC proliferation

**Fig. 3:**
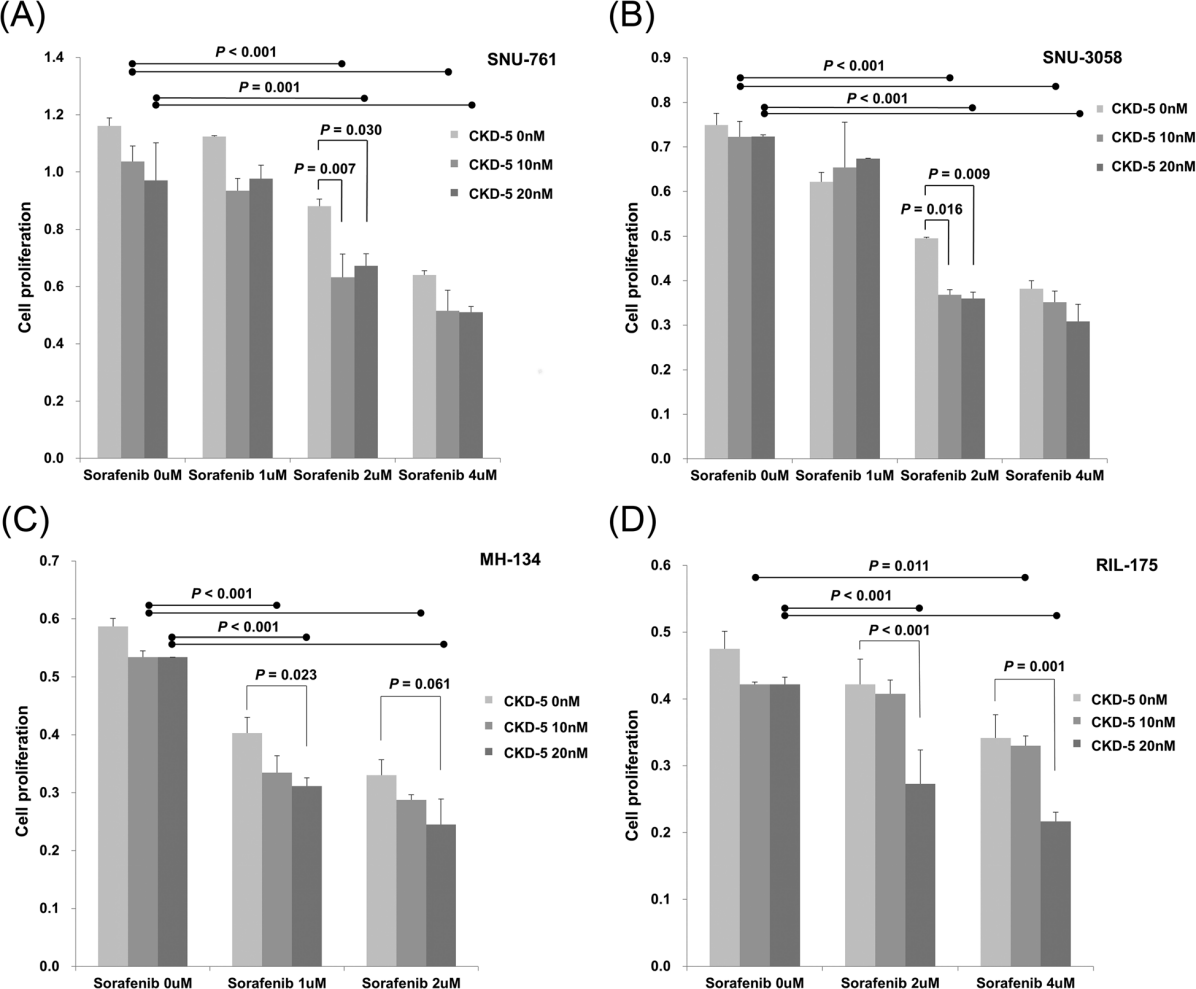
Synergistic anti-tumor effects of CKD-5 in combination with sorafenib on **a** SNU-761, **b** SNU-3058, **c** MH-134 and **d** RIL-175 cells. Combination therapy of CKD-5 with sorafenib significantly decreased HCC cell proliferation compared to CKD-5 or sorafenib monotherapy

The cytotoxicity of co-treatment with CKD-5 and sorafenib was evaluated in SNU-761, SNU-3085, MH-134, and RIL-175 cells. HCC cells were treated with 0–20 nM of CKD-5 and 0–4 μM of sorafenib, followed by MTS assay. Monotherapy with CKD-5 or sorafenib reduced the viability of HCC cells in a dose-dependent manner, and co-treatment with CKD-5 and sorafenib inhibited HCC cell proliferation significantly more than either sorafenib or CKD-5 monotherapy in all four HCC cell lines (Fig. 3). The synergistic anticancer efficacy of the combination therapy with CKD-5 and sorafenib was demonstrated by an excess over Bliss score > 0 in all the investigated HCC cell lines (Supplementary Table 2).

**Fig. 4:**
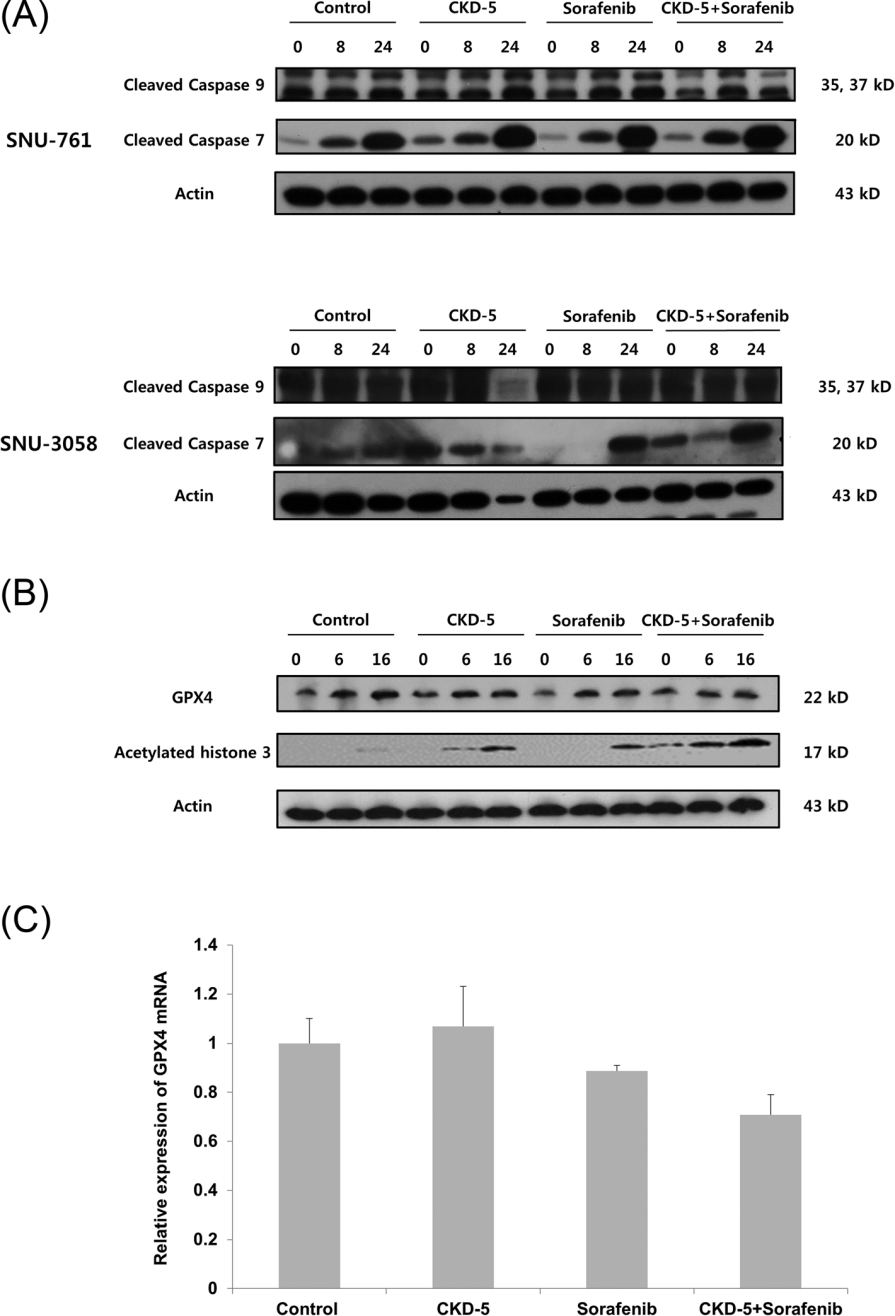
**a** Cleaved caspase-7 and -9 were highly expressed with CKD-5 and sorafenib combination therapy. **b** CKD-5 and sorafenib monotherapy increased acetylated histone H3 levels, which were further increased by the combination treatment with CKD-5 and sorafenib. GPX4 expression was reduced by sorafenib treatment, and further decreased by the combination treatment of sorafenib and CKD-5. **c** mRNA expression of GPX4 was reduced by sorafenib treatment, which was further decreased by the combination treatment of sorafenib and CKD-5

The combination therapy of CKD-5 and sorafenib induced more apoptosis in HCC cells than either CKD-5 or sorafenib monotherapy, showing highly expressed cleaved caspase-7 and -9 after CKD-5 and sorafenib combination treatment (Fig. 4a). Histone acetylation levels increased with CKD-5 and sorafenib monotherapy, and the acetylation level was further increased by the combination treatment with CKD-5 and sorafenib (Fig. 4b). Besides, the protein and mRNA expression of GPX4 was reduced by sorafenib treatment, suggesting that sorafenib induced ferroptosis in HCC cells. The effect was more significant in the combination treatment with sorafenib and CKD-5, suggesting that the induction of ferroptosis by sorafenib was further enhanced by CKD-5 combination (Fig. 4b and c).

### CKD-5 and sorafenib synergistically reduced tumor volume in xenograft mouse models

First, 64 C3H mice implanted with MH-134 HCC cells were divided into 8 groups (control, low-dose CKD-5, high-dose CKD-5, panobinostat, sorafenib, panobinostat plus sorafenib, low-dose CKD-5 plus sorafenib, and high-dose CKD-5 plus sorafenib) and treated with each drug or drug combination accordingly. After 2 weeks of treatment, all the treatment groups exhibited significantly more tumor volume reduction compared with the control group (all *P* < 0.001, Supplement Figure 3). In comparison with the sorafenib group, the CKD-5 groups and the CKD-5 plus sorafenib groups showed significantly decreased tumor volumes (all *P* < 0.001); in contrast, the panobinostat group and the panobinostat plus sorafenib group did not show decreased tumor volumes (both *P* = 1.000).

**Fig. 5:**
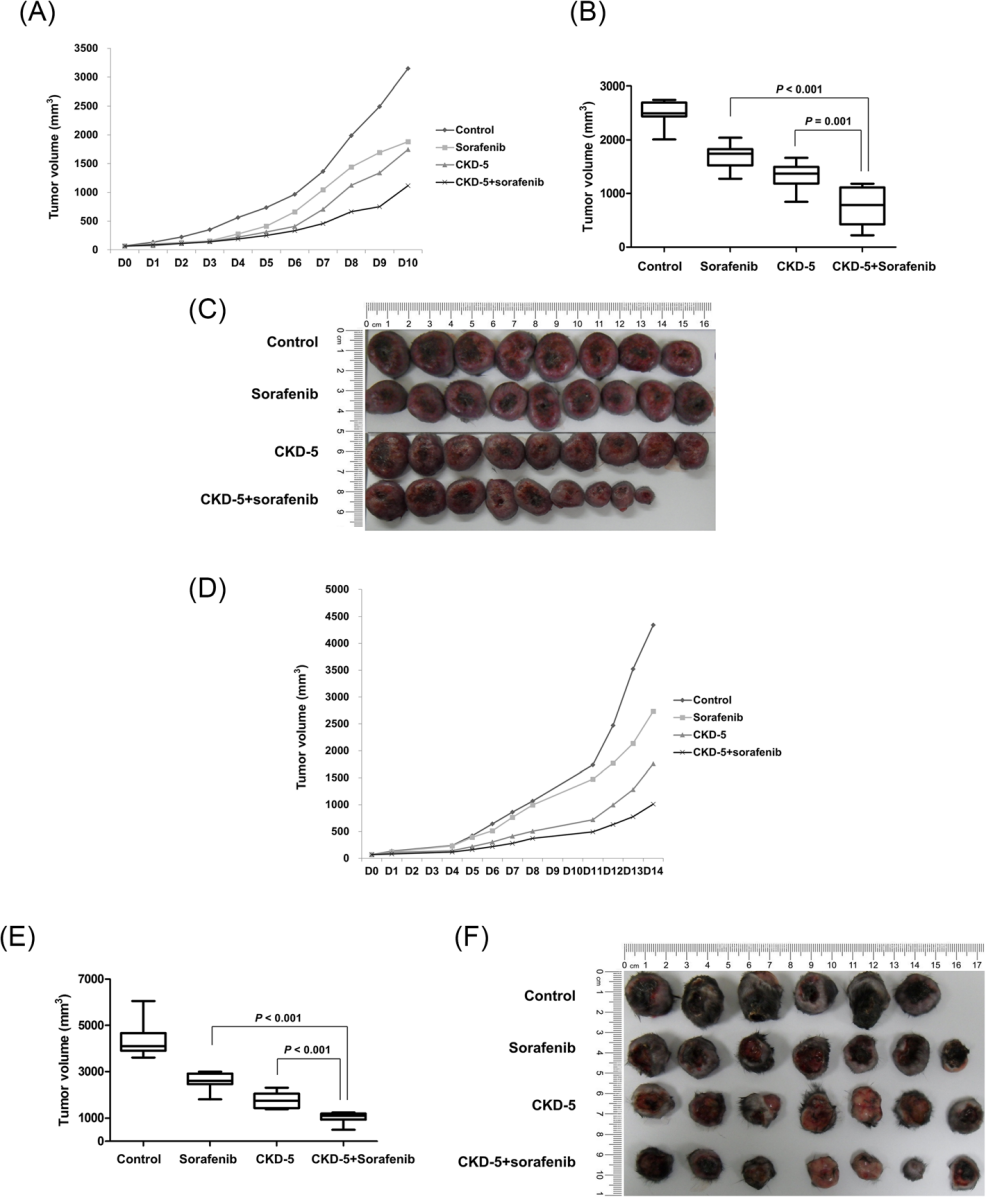
Changes in tumor volume over time after each treatment in (**a**) C3H mice implanted with MH-134 cells and (**d**) C57BL/6 mice implanted with RIL-175 cells. Tumor volumes at the end of each treatment in (**b**) C3H mice implanted with MH-134 cells and (**e**) C57BL/6 mice implanted with RIL-175 cells. Gross specimen of tumors at the end of each treatment in (**c**) C3H mice implanted with MH-134 cells and (**f**) C57BL/6 mice implanted with RIL-175 cells. The numbers of mice assigned to each treatment group are as follows: control (*n* = 8), sorafenib (*n* = 9), CKD-5 (*n* = 9), and CKD-5 plus sorafenib (*n* = 9) in C3H mice, control (*n* = 6), sorafenib (*n* = 7), CKD-5 (*n* = 7), and CKD-5 plus sorafenib (*n* = 7) in C57BL/6 mice. Combination therapy of CKD-5 with sorafenib significantly suppressed tumor growth more than either sorafenib or CKD-5 monotherapy

Based on these results, repeated experiments were performed using the same mouse model with increased numbers in each group to evaluate the synergistic effect of combined CKD-5 and sorafenib. A total of 35 C3H mice implanted with MH-134 HCC cells were assigned to the following 4 groups: control, CKD-5 (60 mg/kg), sorafenib (30 mg/kg), and CKD-5 (60 mg/kg) plus sorafenib (30 mg/kg). After 2 weeks of treatment with each regimen, all the treatment groups showed significantly more tumor volume reduction than the control group (all *P* < 0.001). Additionally, the combination of CKD-5 and sorafenib resulted in significantly less tumor volume than either sorafenib (*P* < 0.001) or CKD-5 (*P* = 0.001) alone (Fig. 5a–c).

In a validation model of C57BL/6 mice implanted with RIL-175 HCC cells, CKD-5 combined with sorafenib significantly inhibited tumor growth more than either sorafenib or CKD-5 (both *P* < 0.001) monotherapy (Fig. 5d–f).

### CKD-5 and sorafenib additively induced apoptosis and reduced vessel density in HCC xenografts

**Fig. 6:**
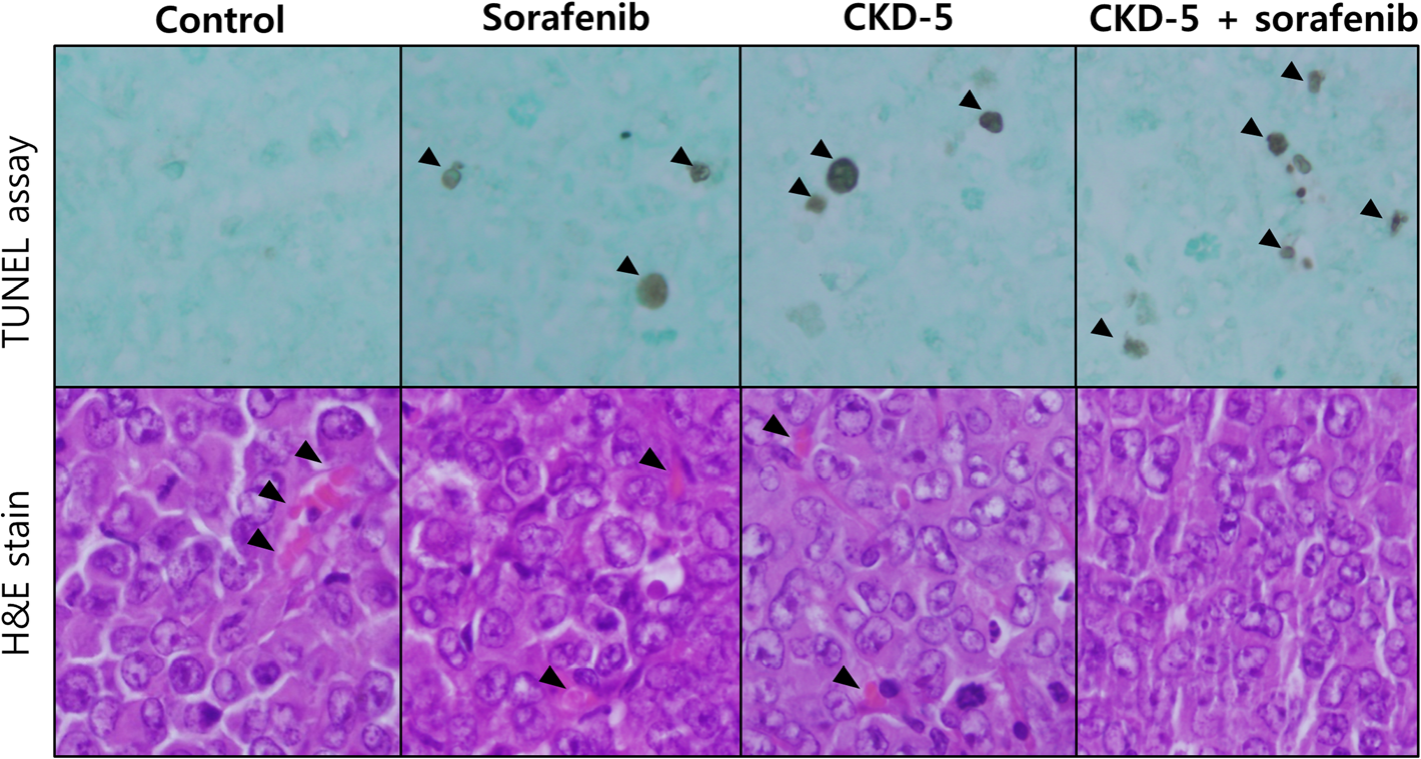
Apoptosis and microvessel density in tumor tissue after each treatment. Combination therapy of CKD-5 with sorafenib markedly increased apoptosis and decreased vessel density compared with CKD-5 or sorafenib monotherapy

To further evaluate apoptosis and angiogenesis in vivo, terminal deoxynucleotidyl transferase dUTP nick end labeling (TUNEL) immunohistochemistry and hematoxylin and eosin (H&E) staining were performed. TUNEL staining revealed a marked increase of apoptotic cells in the tumor tissue of combination treated mice compared to sorafenib or CKD-5 treated mice (Fig. 5). Additionally, the CKD-5 treated mice had decreased microvessel density compared to controls, which was enhanced by combination with sorafenib, resulting in lower vessel density than in the control and sorafenib-treated mice (Fig. 6).

### Combination therapy with CKD-5 and sorafenib was well-tolerated without evidence of renal and hepatic toxicity

The levels of creatinine, alanine transaminase (ALT), and aspartate transaminase (AST) were examined to assess renal and hepatic toxicity of the combination therapy with CKD-5 and sorafenib. The combination treatment group showed significantly lower levels of creatinine and ALT, and the CKD-5 group showed significantly lower levels of ALT and AST, than the control group (Table 1). There were no significant differences in body weight between the treatment groups. These biochemical results demonstrate that there was no renal or hepatotoxicity in the combination therapy group.

**Table 1:** Results of biochemical tests to evaluate renal and hepatic toxicity of sorafenib and CKD-5 treatment

	Control	Sorafenib	CKD-5	CKD-5 + sorafenib
Creatinine (mg/dL)	0.43 ± 0.03	0.41 ± 0.04	0.39 ± 0.02	0.37 ± 0.04^*^
ALT (mg/dL)	221.50 ± 188.65	78.44 ± 45.41^*^	67.75 ± 41.98^*^	70.00 ± 30.71^*^
AST (mg/dL)	578.25 ± 166.24	452.7 ± 77.16	378.88 ± 154.01^*^	412.38 ± 91.83
Weight (g)	25.62 ± 0.94	24.97 ± 0.97	24.76 ± 1.27	24.67 ± 1.17

*ALT* Alanine transaminase, *AST* Aspartate transaminase

^*^ *P* < 0.05 compared to control

Additionally, liver and spleen tissues of sacrificed mice were extracted, and the degree of apoptosis was assessed in both organs by TUNEL and H&E staining to identify toxicities of each treatment. Little apoptosis of the liver and spleen tissues was detected in all treatment groups (Supplementary Figure 4).

## Discussion

Sorafenib was the first approved targeted agent in advanced HCC, achieving a significantly prolonged median survival time of 10.7 months in the treatment group compared to 7.9 months in a control group that received supportive care only [2]. However, the need for a new therapeutic agent or a combination agent with sorafenib has emerged due to the limited efficacy of sorafenib monotherapy [3]. Many clinical trials investigating effective anti-tumor agents in combination with sorafenib have failed to prove the superiority of combination therapy over sorafenib monotherapy [34]. In response to this need, we investigated the efficacy of the combination of sorafenib and CKD-5, a novel pan-HDACI that was found to have an acceptable safety profile in a phase I study involving patients with multiple myeloma [19].

In this study, we demonstrated the anticancer effect of CKD-5 in HCC, which was synergistically enhanced by sorafenib. In vitro, CKD-5 monotherapy reduced cell viability and induced HCC cell apoptosis, and peripherin was involved in CKD-5-mediated HCC apoptosis. Other known HDACI-related mediators such as P21 and HSP90 were not associated with CKD-5 treatment. When combined with sorafenib, CKD-5 synergistically inhibited HCC proliferation. In vivo, CKD-5 alone decreased tumor volume in treated mice compared to controls, and the combination of CKD-5 and sorafenib synergistically reduced tumor volume in the mouse models. There were no demonstrable major organ toxicities. These results indicate the potential efficacy and safety of combination therapy with CKD-5 and sorafenib.

Generally, HDACIs manifest a direct anti-cancer effect by hyperacetylating histone or non-histone proteins. Several HDACIs with proven clinical efficacy for cancer treatment have received US FDA approval. Vorinostat and romidepsin for cutaneous T-cell lymphoma [35, 36], belinostat for relapsed or refractory peripheral T-cell lymphoma [37], and panobinostat for multiple myeloma [38] were sequentially approved. Although the anticancer effects of HDACIs are largely seen in hematologic malignancies, they have also been demonstrated in preclinical studies of solid cancers, particularly when administered in combination with various other treatments: with lapatinib in colon cancer [39], irradiation in non-small cell lung cancer [40], and sorafenib in HCC [15]. Further, in a recently reported case of panobinostat treatment in combination with sorafenib, a patient with advanced stage HCC showed a partial response [41]. We used panobinostat as a combination agent with sorafenib to compare its anticancer efficacy with CKD-5. As a result, CKD-5 showed higher anticancer efficacy than panobinostat when combined with sorafenib. This result demonstrates that anti-cancer effects of various HDACIs vary by tumor type and CKD-5 may have a greater efficacy in HCC than panobinostat.

We have identified peripherin as a possible mechanism of anticancer activity of CKD-5 by cDNA microarray analysis and it was validated with real-time PCR. Peripherin is a protein-coding gene that encodes a type III intermediate filament protein found in the neurons of the peripheral nervous system [42]. Its function and related diseases have not been fully understood, and there are only reports confined to the nervous system, such as axonal regrowth [43] or amyptrophic lateral sclerosis [44–46]. Recently, Revill et al. [47] have reported peripherin as one of 13 novel tumor suppressor candidate genes in HCC, although two other genes were finally selected after subsequent validation. Enrichr analysis showed that peripherin was a negative target of the polycomb repressive complex 2 (PRC2) [48]. PRC2 is one of the two classes of polycomb-group proteins, which has histone methyltransferase activity and primarily methylates histone H3 [49, 50]. PRC2 is known to interact with HDACs in transcriptional silencing and is related to tumor suppressor loss [51, 52]. These studies suggest that CKD-5-induced peripherin may act as a tumor suppressor by repressing PRC2.

A recent study has reported that inhibition of HDAC is one of the anticancer activities of sorafenib, and upregulated HDAC activity may contribute to the development of sorafenib resistance [53]. In this study, we demonstrated that both CKD-5 and sorafenib increased histone acetylation, which was further enhanced with combination of CKD-4 and sorafenib. These findings suggest that CKD-5, a novel HDACi, enhanced the anticancer activity of sorafenib by amplifying the HDAC inhibitory activity of sorafenib. Several reports showed that the anticancer efficacy of sorafenib was enhanced or restored by HDAC inhibition with various agents. Statins have been reported to assist in overcoming the hypoxic resistance of HCC cells to sorafenib by inhibition of HDAC [54]. Methotrexate showed synergistic anticancer effects in combination with sorafenib through HDAC inhibition [55]. Panobinostat, the first reported HDACI, showed synergistic anticancer efficacy with sorafenib in a preclinical study and a case report [15, 41]. In the present study, CKD-5 demonstrated superior anticancer efficacy compared to panobinostat in combination with sorafenib.

Ferroptosis is a recently recognized mechanism of regulated cell death that is distinct from apoptosis and necroptosis [56]. Glutathione peroxidase 4 (GPX4) is known as the central regulator of ferroptosis; a decrease in GPX4 expression is a signal of ferroptosis Ferroptosis is involved in various diseases, including cancer [57], and sorafenib induces ferroptosis in HCC [58]. This was also confirmed in our study. Furthermore, we found that the induction of ferroptosis was enhanced by the combination of sorafenib with CKD-5.

There are several limitations in this study. Although the synergistic anticancer effect of combination therapy with CKD-5 and sorafenib was demonstrated using an appropriate statistical model, the synergistic efficacy was less significant in vitro than in vivo. The synergistic effects of CKD-5 and sorafenib may be achieved by modulating the tumor microenvironement as well as the cancer cells directly. Second, the role of peripherin in the synergistic anticancer effect of CKD-5 and sorafenib has not been investigated in this study. Further studies are needed to understand the mechanism of such synergism.

## Conclusion

In conclusion, CKD-5 has direct anticancer effects and it has synergistic effects on HCC suppression when combined with sorafenib. Our results also suggest that CKD-5 may be superior to panobinostat in treating HCC.

## Supplementary information

**Additional file 1: Table S1.** A list of genes up-regulated with fold change ≥2 in cDNA microarray analysis. **Table S2.** Growth inhibition rates of each treatment. **Figure S1.** The cytotoxic efficacy of CKD-5 and panobinostat in SNU-761 cells. Both panobinostat and CKD-5 treatment reduced cell proliferation, and the cytotoxic efficacy was more potent in CKD-5 than panobinostat especially when combined with Sorafenib. **Figure S2.** Results of cDNA microarray assay. (A) The number of up- or down-regulated probes filtered by *P*-value and various fold changes. (B) A scatter plot of expression level between the control and HDACi-treated samples. (C) Hierarchical clustering heatmap. (D-F) Gene-enrichment and functional annotation analysis using DAVID based on Gene Ontology database. **Figure S3.** Changes in tumor volume over time after each treatment in a model of C3H mouse implanted with MH-134 cells. Combination therapy of high dose CKD-5 with sorafenib significantly suppressed tumor growth more than any other treatment. **Figure S4.** The degree of apoptosis in (A) liver and (B) spleen tissue assessed by TUNEL and H&E staining. Little apoptosis of the liver and spleen tissue was detected in all treatment groups.

## Data Availability

The datasets used and/or analyzed during the current study are available from the corresponding author on reasonable request.

## Abbreviations

HCC: Hepatocellular carcinoma
HDACI: Histone deacetylase inhibitor
FBS: Fetal bovine serum
siRNA: Small interfering RNA
MTS: 3,4-(5-dimethylthiazol-2-yl)-5-(3-carboxymethoxyphenyl)-2-(4-sulfo-phenyl)-2H-tetrazolium salt
RNA: Ribonucleic acid
PCR: Polymerase chain reaction
cDNA: Complementary deoxyribonucleic acid
GAPDH: Glyceraldehyde-3-phosphate dehydrogenase

## References

[1] Bray F, Ferlay J, Soerjomataram I, Siegel RL, Torre LA, Jemal A (2018). Global cancer statistics 2018: GLOBOCAN estimates of incidence and mortality worldwide for 36 cancers in 185 countries. CA Cancer J Clin.

[2] Cheng AL, Kang YK, Chen Z, Tsao CJ, Qin S, Kim JS, Luo R, Feng J, Ye S, Yang TS (2009). Efficacy and safety of sorafenib in patients in the Asia-Pacific region with advanced hepatocellular carcinoma: a phase III randomised, double-blind, placebo-controlled trial. Lancet Oncol.

[3] Gauthier A, Ho M (2013). Role of sorafenib in the treatment of advanced hepatocellular carcinoma: an update. Hepatol Res.

[4] Geschwind JF, Kudo M, Marrero JA, Venook AP, Chen XP, Bronowicki JP, Dagher L, Furuse J, Ladron de Guevara L, Papandreou C (2016). TACE treatment in patients with Sorafenib-treated Unresectable hepatocellular carcinoma in clinical practice: final analysis of GIDEON. Radiology.

[5] Peng Z, Chen S, Wei M, Lin M, Jiang C, Mei J, Li B, Wang Y, Li J, Xie X (2018). Advanced recurrent hepatocellular carcinoma: treatment with Sorafenib alone or in combination with Transarterial chemoembolization and radiofrequency ablation. Radiology.

[6] Cosgrove DP, Reyes DK, Pawlik TM, Feng AL, Kamel IR, Geschwind JF (2015). Open-label single-arm phase II trial of Sorafenib therapy with drug-eluting bead Transarterial chemoembolization in patients with Unresectable hepatocellular carcinoma: clinical results. Radiology.

[7] Abou-Alfa GK, Johnson P, Knox JJ, Capanu M, Davidenko I, Lacava J, Leung T, Gansukh B, Saltz LB (2010). Doxorubicin plus sorafenib vs doxorubicin alone in patients with advanced hepatocellular carcinoma: a randomized trial. JAMA.

[8] Patt Y, Rojas-Hernandez C, Fekrazad HM, Bansal P, Lee FC (2017). Phase II trial of Sorafenib in combination with Capecitabine in patients with hepatocellular carcinoma: INST 08-20. Oncologist.

[9] Naqi N, Ahmad S, Murad S, Khattak J (2014). Efficacy and safety of sorafenib-gemcitabine combination therapy in advanced hepatocellular carcinoma: an open-label phase II feasibility study. Hematol Oncol Stem Cell Ther.

[10] Abou-Alfa GK, Yen CJ, Hsu CH, O'Donoghue J, Beylergil V, Ruan S, Pandit-Taskar N, Gansukh B, Lyashchenko SK, Ma J (2017). Phase Ib study of codrituzumab in combination with sorafenib in patients with non-curable advanced hepatocellular carcinoma (HCC). Cancer Chemother Pharmacol.

[11] Turcios L, Vilchez V, Acosta LF, Poyil P, Butterfield DA, Mitov M, Marti F, Gedaly R (2017). Sorafenib and FH535 in combination act synergistically on hepatocellular carcinoma by targeting cell bioenergetics and mitochondrial function. Dig Liver Dis.

[12] Duffy AG, Ma C, Ulahannan SV, Rahma OE, Makarova-Rusher O, Cao L, Yu Y, Kleiner DE, Trepel J, Lee MJ (2017). Phase I and preliminary phase II study of TRC105 in combination with Sorafenib in hepatocellular carcinoma. Clin Cancer Res.

[13] Prince HM, Bishton MJ, Harrison SJ (2009). Clinical studies of histone deacetylase inhibitors. Clin Cancer Res.

[14] West AC, Johnstone RW (2014). New and emerging HDAC inhibitors for cancer treatment. J Clin Invest.

[15] Lachenmayer A, Toffanin S, Cabellos L, Alsinet C, Hoshida Y, Villanueva A, Minguez B, Tsai HW, Ward SC, Thung S (2012). Combination therapy for hepatocellular carcinoma: additive preclinical efficacy of the HDAC inhibitor panobinostat with sorafenib. J Hepatol.

[16] Lee C, Ahn K-S, Jung WJ, Koh Y, Kim HJ, Lee HJ, Yoon H-J, Yoon S-S (2014). Abstract 1695: CKD-581, a novel histone deacetylase inhibitor, synergistically enhances Bortezomib cytotoxicity in multiple myeloma cells. Cancer Res.

[17] Kim MJ, Lee CS, Lee DH, Yang HM, Lim IT, Bae DI, Choe YJ, Kim DH, Kim SK, Lee SS (2011). 9208 POSTER activity of CKD-581, histone Deacetylase inhibitor, in cutaneous T-cell lymphoma models. Eur J Cancer.

[18] Kim MJ, Lee CS, Han BH, Yang H-M, Lee KJ, Kim S-M, Oh HT, Lim I-T, Shin H, Bae D (2010). abstract 5435: a novel HDAC inhibitor, CKD-581, demonstrates a potent <em>in vivo</em> efficacy in various human tumor xenograft models. Cancer Res.

[19] Cho H, Yoon DH, Kim KP, Bae KS, Kim WS, Eom HS, Kim JS, Hong JY, Kim SJ, Lee H (2018). Phase I study of CKD-581, a pan-histone deacetylase inhibitor, in patients with lymphoma or multiple myeloma refractory to standard therapy. Investig New Drugs.

[20] Nakabayashi H, Taketa K, Miyano K, Yamane T, Sato J (1982). Growth of human hepatoma cells lines with differentiated functions in chemically defined medium. Cancer Res.

[21] Lee JH, Ku JL, Park YJ, Lee KU, Kim WH, Park JG (1999). Establishment and characterization of four human hepatocellular carcinoma cell lines containing hepatitis B virus DNA. World J Gastroenterol.

[22] Yamashita YI, Shimada M, Hasegawa H, Minagawa R, Rikimaru T, Hamatsu T, Tanaka S, Shirabe K, Miyazaki JI, Sugimachi K (2001). Electroporation-mediated interleukin-12 gene therapy for hepatocellular carcinoma in the mice model. Cancer Res.

[23] Park JG, Lee JH, Kang MS, Park KJ, Jeon YM, Lee HJ, Kwon HS, Park HS, Yeo KS, Lee KU (1995). Characterization of cell lines established from human hepatocellular carcinoma. Int J Cancer.

[24] Kim KR, Moon HE, Kim KW (2002). Hypoxia-induced angiogenesis in human hepatocellular carcinoma. J Mol Med (Berl).

[25] Cho Y, Cho EJ, Lee JH, Yu SJ, Kim YJ, Kim CY, Yoon JH (2016). Fucoidan-induced ID-1 suppression inhibits the in vitro and in vivo invasion of hepatocellular carcinoma cells. Biomed Pharmacother.

[26] Llovet JM, Chen Y, Wurmbach E, Roayaie S, Fiel MI, Schwartz M, Thung SN, Khitrov G, Zhang W, Villanueva A (2006). A molecular signature to discriminate dysplastic nodules from early hepatocellular carcinoma in HCV cirrhosis. Gastroenterology.

[27] Saijo H, Tatsumi N, Arihiro S, Kato T, Okabe M, Tajiri H, Hashimoto H (2015). Microangiopathy triggers, and inducible nitric oxide synthase exacerbates dextran sulfate sodium-induced colitis. Lab Investig.

[28] Clarke WT, Edwards B, McCullagh KJ, Kemp MW, Moorwood C, Sherman DL, Burgess M, Davies KE (2010). Syncoilin modulates peripherin filament networks and is necessary for large-calibre motor neurons. J Cell Sci.

[29] Li X, Duan L, Yuan S, Zhuang X, Qiao T, He J (2019). Ferroptosis inhibitor alleviates radiation-induced lung fibrosis (RILF) via down-regulation of TGF-beta1. J Inflamm (Lond).

[30] Nakatani S, Iwagaki H, Okabayashi T, Matsubara N, Isozaki H, Takakura N, Horimi T, Tanaka N (1999). Effects of streptococcal preparation OK-432 on cytokine induction in spleen and tumour tissues of mice bearing MH-134 tumour cells. J Int Med Res.

[31] Orci LA, Lacotte S, Delaune V, Slits F, Oldani G, Lazarevic V, Rossetti C, Rubbia-Brandt L, Morel P, Toso C (2018). Effects of the gut-liver axis on ischaemia-mediated hepatocellular carcinoma recurrence in the mouse liver. J Hepatol.

[32] Hou H, Lariviere JP, Demidenko E, Gladstone D, Swartz H, Khan N (2009). Repeated tumor pO(2) measurements by multi-site EPR oximetry as a prognostic marker for enhanced therapeutic efficacy of fractionated radiotherapy. Radiother Oncol.

[33] Liu Q, Yin X, Languino LR, Altieri DC (2018). Evaluation of drug combination effect using a bliss independence dose-response surface model. Stat Biopharm Res.

[34] Kudo M (2017). Systemic Therapy for Hepatocellular Carcinoma: 2017 Update. Oncology.

[35] Olsen EA, Kim YH, Kuzel TM, Pacheco TR, Foss FM, Parker S, Frankel SR, Chen C, Ricker JL, Arduino JM (2007). Phase IIb multicenter trial of vorinostat in patients with persistent, progressive, or treatment refractory cutaneous T-cell lymphoma. J Clin Oncol.

[36] Whittaker SJ, Demierre MF, Kim EJ, Rook AH, Lerner A, Duvic M, Scarisbrick J, Reddy S, Robak T, Becker JC (2010). Final results from a multicenter, international, pivotal study of romidepsin in refractory cutaneous T-cell lymphoma. J Clin Oncol.

[37] Lee HZ, Kwitkowski VE, Del Valle PL, Ricci MS, Saber H, Habtemariam BA, Bullock J, Bloomquist E, Li Shen Y, Chen XH (2015). FDA approval: Belinostat for the treatment of patients with relapsed or refractory peripheral T-cell lymphoma. Clin Cancer Res.

[38] Laubach JP, Moreau P, San-Miguel JF, Richardson PG (2015). Panobinostat for the treatment of multiple myeloma. Clin Cancer Res.

[39] LaBonte MJ, Wilson PM, Fazzone W, Russell J, Louie SG, El-Khoueiry A, Lenz HJ, Ladner RD (2011). The dual EGFR/HER2 inhibitor lapatinib synergistically enhances the antitumor activity of the histone deacetylase inhibitor panobinostat in colorectal cancer models. Cancer Res.

[40] Geng L, Cuneo KC, Fu A, Tu T, Atadja PW, Hallahan DE (2006). Histone deacetylase (HDAC) inhibitor LBH589 increases duration of gamma-H2AX foci and confines HDAC4 to the cytoplasm in irradiated non-small cell lung cancer. Cancer Res.

[41] Knieling F, Waldner MJ, Goertz RS, Strobel D (2012). Quantification of dynamic contrast-enhanced ultrasound in HCC: prediction of response to a new combination therapy of sorafenib and panobinostat in advanced hepatocellular carcinoma. BMJ Case Rep.

[42] Portier MM, de Nechaud B, Gros F (1983). Peripherin, a new member of the intermediate filament protein family. Dev Neurosci.

[43] Oblinger MM, Wong J, Parysek LM (1989). Axotomy-induced changes in the expression of a type III neuronal intermediate filament gene. J Neurosci.

[44] He CZ, Hays AP (2004). Expression of peripherin in ubiquinated inclusions of amyotrophic lateral sclerosis. J Neurol Sci.

[45] Gros-Louis F, Lariviere R, Gowing G, Laurent S, Camu W, Bouchard JP, Meininger V, Rouleau GA, Julien JP (2004). A frameshift deletion in peripherin gene associated with amyotrophic lateral sclerosis. J Biol Chem.

[46] Leung CL, He CZ, Kaufmann P, Chin SS, Naini A, Liem RK, Mitsumoto H, Hays AP (2004). A pathogenic peripherin gene mutation in a patient with amyotrophic lateral sclerosis. Brain Pathol.

[47] Revill K, Wang T, Lachenmayer A, Kojima K, Harrington A, Li J, Hoshida Y, Llovet JM, Powers S (2013). Genome-wide methylation analysis and epigenetic unmasking identify tumor suppressor genes in hepatocellular carcinoma. Gastroenterology.

[48] Chen EY, Tan CM, Kou Y, Duan Q, Wang Z, Meirelles GV, Clark NR, Ma'ayan A (2013). Enrichr: interactive and collaborative HTML5 gene list enrichment analysis tool. BMC Bioinformatics.

[49] Chase A, Cross NC (2011). Aberrations of EZH2 in cancer. Clin Cancer Res.

[50] Yoo KH, Hennighausen L (2012). EZH2 methyltransferase and H3K27 methylation in breast cancer. Int J Biol Sci.

[51] Kuzmichev A, Nishioka K, Erdjument-Bromage H, Tempst P, Reinberg D (2002). Histone methyltransferase activity associated with a human multiprotein complex containing the enhancer of Zeste protein. Genes Dev.

[52] van der Vlag J, Otte AP (1999). Transcriptional repression mediated by the human polycomb-group protein EED involves histone deacetylation. Nat Genet.

[53] Liu TP, Hong YH, Yang PM (2017). In silico and in vitro identification of inhibitory activities of sorafenib on histone deacetylases in hepatocellular carcinoma cells. Oncotarget.

[54] Zhou TY, Zhuang LH, Hu Y, Zhou YL, Lin WK, Wang DD, Wan ZQ, Chang LL, Chen Y, Ying MD (2016). Inactivation of hypoxia-induced YAP by statins overcomes hypoxic resistance tosorafenib in hepatocellular carcinoma cells. Sci Rep.

[55] Lee D, Xu IM, Chiu DK, Lai RK, Tse AP, Lan Li L, Law CT, Tsang FH, Wei LL, Chan CY (2017). Folate cycle enzyme MTHFD1L confers metabolic advantages in hepatocellular carcinoma. J Clin Invest.

[56] Dixon SJ, Lemberg KM, Lamprecht MR, Skouta R, Zaitsev EM, Gleason CE, Patel DN, Bauer AJ, Cantley AM, Yang WS (2012). Ferroptosis: an iron-dependent form of nonapoptotic cell death. Cell.

[57] Masaldan S, Bush AI, Devos D, Rolland AS, Moreau C (2019). Striking while the iron is hot: Iron metabolism and ferroptosis in neurodegeneration. Free Radic Biol Med.

[58] Lachaier E, Louandre C, Godin C, Saidak Z, Baert M, Diouf M, Chauffert B, Galmiche A (2014). Sorafenib induces ferroptosis in human cancer cell lines originating from different solid tumors. Anticancer Res.

