# Supplementary material for: CKD-5, a novel pan-histone deacetylase inhibitor, synergistically enhances the efficacy of sorafenib for hepatocellular carcinoma
Source: BMC Cancer. 2020 Oct 15;20:1001. doi: 10.1186/s12885-020-07471-3 (PMC7559883; doi:10.1186/s12885-020-07471-3)
Supplement: Supplementary file 1 — Additional file 1: Table S1. A list of genes up-regulated with fold change ≥2 in cDNA microarray analysis. Table S2. Growth inhibition rates of each treatment. Figure S1. The cytotoxic efficacy of CKD-5 and panobinostat in SNU-761 cells. Both panobinostat and CKD-5 treatment reduced cell proliferation, and the cytotoxic efficacy was more potent in CKD-5 than panobinostat especially when combined with Sorafenib. Figure S2. Results of cDNA microarray assay. (A) The number of up- or down-regulated probes filtered by P-value and various fold changes. (B) A scatter plot of expression level between the control and HDACi-treated samples. (C) Hierarchical clustering heatmap. (D-F) Gene-enrichment and functional annotation analysis using DAVID based on Gene Ontology database. Figure S3. Changes in tumor volume over time after each treatment in a model of C3H mouse implanted with MH-134 cells. Combination therapy of high dose CKD-5 with sorafenib significantly suppressed tumor growth more than any other treatment. Figure S4. The degree of apoptosis in (A) liver and (B) spleen tissue assessed by TUNEL and H&E staining. Little apoptosis of the liver and spleen tissue was detected in all treatment groups. [file 12885_2020_7471_MOESM1_ESM.zip › CKD_Supplement_Chang et al_revisedR2.docx]

**Supplementary Tables**

**Supplementary Table 1.** A list of genes up-regulated with fold change ≥ 2 in cDNA microarray analysis

| CRABP2 NPPB AIF1L PRPH PTH2 CA4 UCA1 LOC100129681 FLJ35767 CGA PGC CNFN C15orf48 IFI6 MAGEB2 SNCA GMFG COL1A2 TMEM158 ULBP2 PODXL HLA-DMB MFGE8 COL1A2 CLCNKA CLCNKA HLA-B ATP8B3 NELL2 OAS3 ARMCX2 MT1G MX1 TMSB15A PPP1R1A HAMP NDRG4 ISG15 MT2A TMEM151A IFI6 GAGE2B CD79B COL11A1 IRF9 CDC42EP5 KLRC2 UPK1A EFHD1 ELOVL4 SYT11 MUC13 VCX2 CRIP2 VCX2 SERPINE2 CT45A4 CPE KREMEN2 ACVRL1 IL8 CGB1 NPAS1 TPD52L1 NTS IL1R2 LIPG ATP6V0E2 TIMP2 KCNS1 HEY1 MAGEA9B BHMT IL13RA2 EPSTI1 COL8A2 ULBP1 CD14 PARM1 HLA-E IGFBP4 CREG1 CT45A4 VCX3A CDKN1A MT1A IL8 S100A9 PPP2R2B HLA-DRA MAGEA4 PTGER4 FXYD5 DLL3 ADAP2 VCX2 FAM125A LOC124220 PTPLA MTMR11 VCX3A PRNP INHBE GLIPR2 NLRP7 VCX2 ICAM2 FSTL3 TNFSF9 C12orf39 MT1H OLFML2A ADAM19 CCNB3 CYFIP2 SLPI CPA4 TESC LOC401720 OLR1 RGS17 SLC7A8 CENTA1 WAS CCDC151 LOC346887 CRLF1 GAD1 EDN1 C1QL4 ANKRD24 KIF5C VCX-C APLP1 SERPINI1 PLAT SERPINI1 CYR61 IFIT1 RGS22 RGS10 LIPG SMPDL3B HERC5 GPSM1 ATF3 LMTK3 DEFB1 PRNP FHL1 QPCT TIMP4 CT45A4 LOC100134361 TMEM145 IGSF11 GPR160 MSX1 KCNK6 CYP2S1 SOCS2 ROBO3 ANXA1 PCDH20 CPT1C MGC57346 PAGE1 TMEM59L CLTB GRM3 ZNF280A SAMD11 CD47 FYN DUSP1 VCX MCD1 TMEM154 MGC39900 LGMN ALPK2 FLRT2 C4orf49 HIS T1H2BK SLC1A3 FAM84B IFIH1 NRIP3 ST6GALNAC3 C11orf70 FBLN2 PRSS23 NRGN BEX5 ARL14 OC342979 C4orf49 SOX18 PRIC285 HIST1H1C APOD LUM RBP7 PRSS35 PLEK2 CD24 REEP2 CLDN11 CCDC74B HSD17B6 BIRC3 UACA MT1E MAGEB6B PI3 SAA1 CADM1 SRPX ABCB1 ELMO1 NCF2 SSX1 GJA1 NLF2 XAGE2B FAM19A2 HIST1H2BD CLDN18 LOC647784 PMP22 F2RL1 SAA2 CAND2 S100A3 LEPREL1 TNFRSF12A LOC338758 PDGFD KRT10 MTE FAM19A2 CD9 OSAP LGMN PARP9 LPHN1 FEZ1 COL8A2 PCDHB6 SMPDL3B RFTN1 XAGE1B SEMA4F MGC39900 OAS1 CFD MT1X CAV1 TPD52L1 SOCS2 MAGEH1 C1orf61 LOC653110 RNASEL HPSE BEX2 LYN HERC6 PCSK1N CER1 MT1F TMEM154 PLSCR1 MOXD1 HIST1H4H TFPI2 RASIP1 FZD9 CCNB3 CT45A5 NOS3 CPT1A NEO1 DDX60 SMAD7 MYH6 SLC2A10 RNASEL TUSC3 LIPA IRX3 F2RL1 SSX4B ENO3 S100A4 KLRC3 RABAC1 BIRC3 TRK1 LOC645558 HES4 CTSL1 NLRP2 NUAK1 GLT8D2 PLAC8 SCPEP1 CNIH2 CT45A5 NNMT LOC100129668 ITGA2 CDCP1 ACSL5 TMEM125 SCAMP5 C11orf70 LOC92249 TGFB2 TSPAN8 ONECUT2 DKK3 SYT1 OSGIN2 TAP1 LOC387882 TRIM36 NLRP7 TUBB2B TRIB1 SILV FLJ22184 FABP5L2 CAV2 CD83 TSPAN7 SMPDL3B HEG1 GOLM1 FAM108C1 SLC25A24 COL24A1 C14orf72 FABP5 SDF2L1 SEC11C IDS GEM RASL10A HIST1H2BK CYB561 CHSY1 PAGE2 CDS1 PLAC8 SLC46A3 LOC732150 PAGE2B FAM104B BHLHB2 DPYSL3 CAPRIN2 EOMES CYB561 |
| --- |

**Supplementary Table 2.** Growth inhibition rates of each treatment.

| Cell lines | Control | CKD-5 | Sorafenib | CKD-5+Sorafenib | | Excess over Bliss score† |
| --- | --- | --- | --- | --- | --- | --- |
|  |  |  |  | Predicted* | Observed |  |
| SNU-761 | 0.00 | 0.16 | 0.45 | 0.54 | 0.56 | 0.02 |
| SNU-3058 | 0.00 | 0.03 | 0.49 | 0.51 | 0.59 | 0.08 |
| MH-134 | 0.00 | 0.09 | 0.44 | 0.49 | 0.58 | 0.09 |
| RIL-175 | 0.00 | 0.11 | 0.28 | 0.36 | 0.54 | 0.18 |

*Predicted cell inhibition rate which was calculated assuming drug independence according to Bliss: M_A_ + M_B_ - M_A_M_B_ = M_AB_

†Excess over Bliss score = y_ab_ - ŷ_ab_

M, mortality; ŷ, predicted response; y, observed response

**Supplementary Figure Legends**

**Supplementary Figure 1.** The cytotoxic efficacy of CKD-5 and panobinostat in SNU-761 cells. Both panobinostat and CKD-5 treatment reduced cell proliferation, and the cytotoxic efficacy was more potent in CKD-5 than panobinostat especially when combined with Sorafenib

**Supplementary Figure 2.** Results of cDNA microarray assay. (A) The number of up- or down-regulated probes filtered by P-value and various fold changes. (B) A scatter plot of expression level between the control and HDACi-treated samples. (C) Hierarchical clustering heatmap. (D-F) Gene-enrichment and functional annotation analysis using DAVID based on Gene Ontology database

**Supplementary Figure 3.** Changes in tumor volume over time after each treatment in a model of C3H mouse implanted with MH-134 cells. Combination therapy of high dose CKD-5 with sorafenib significantly suppressed tumor growth more than any other treatment.

**Supplementary Figure 4.** The degree of apoptosis in (A) liver and (B) spleen tissue assessed by TUNEL and H&E staining. Little apoptosis of the liver and spleen tissue was detected in all treatment groups
