# Supplementary figures and images for: CKD-5, a novel pan-histone deacetylase inhibitor, synergistically enhances the efficacy of sorafenib for hepatocellular carcinoma
Source: BMC Cancer. 2020 Oct 15;20:1001. doi: 10.1186/s12885-020-07471-3 (PMC7559883; doi:10.1186/s12885-020-07471-3)

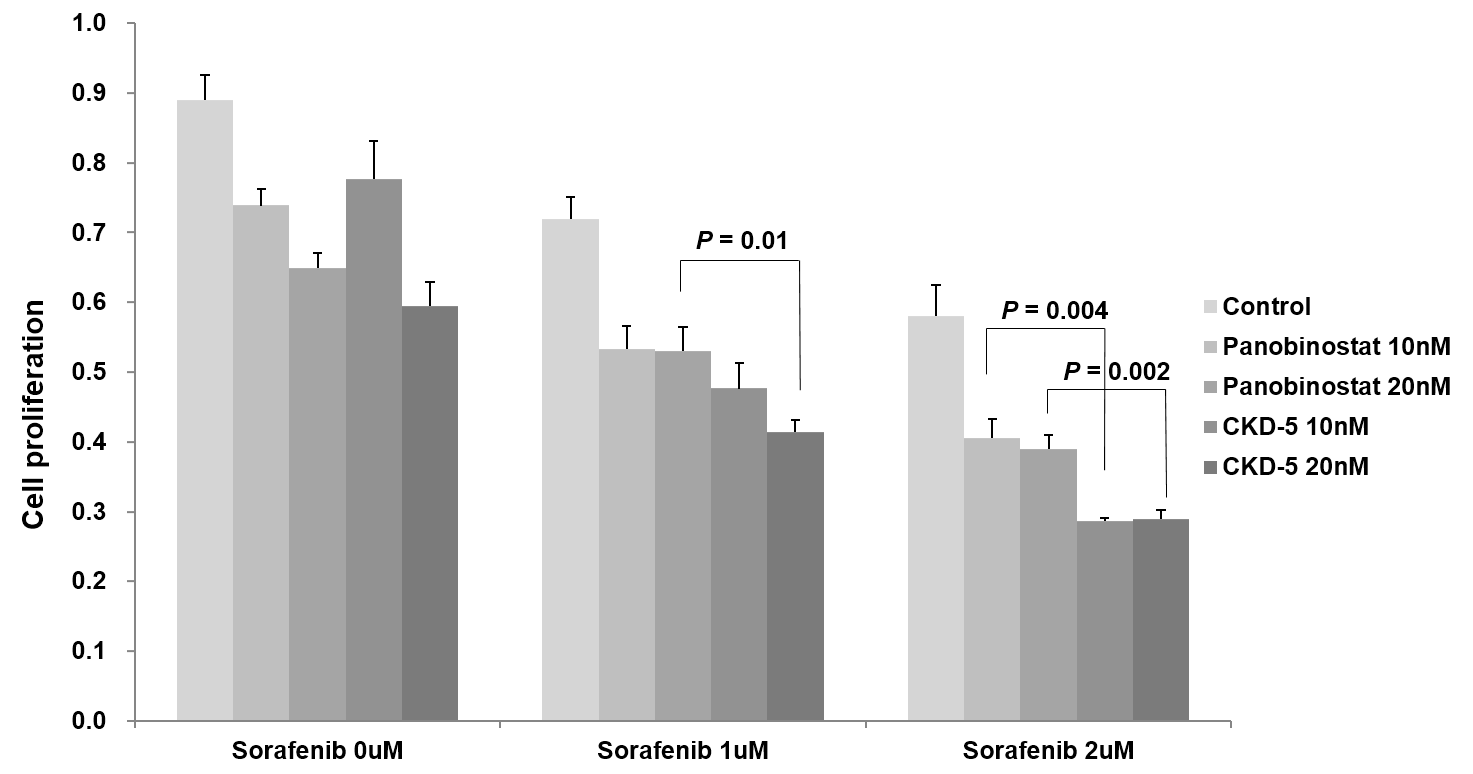

Supplement: Supplementary file 1 — Additional file 1: Table S1. A list of genes up-regulated with fold change ≥2 in cDNA microarray analysis. Table S2. Growth inhibition rates of each treatment. Figure S1. The cytotoxic efficacy of CKD-5 and panobinostat in SNU-761 cells. Both panobinostat and CKD-5 treatment reduced cell proliferation, and the cytotoxic efficacy was more potent in CKD-5 than panobinostat especially when combined with Sorafenib. Figure S2. Results of cDNA microarray assay. (A) The number of up- or down-regulated probes filtered by P-value and various fold changes. (B) A scatter plot of expression level between the control and HDACi-treated samples. (C) Hierarchical clustering heatmap. (D-F) Gene-enrichment and functional annotation analysis using DAVID based on Gene Ontology database. Figure S3. Changes in tumor volume over time after each treatment in a model of C3H mouse implanted with MH-134 cells. Combination therapy of high dose CKD-5 with sorafenib significantly suppressed tumor growth more than any other treatment. Figure S4. The degree of apoptosis in (A) liver and (B) spleen tissue assessed by TUNEL and H&E staining. Little apoptosis of the liver and spleen tissue was detected in all treatment groups. [file 12885_2020_7471_MOESM1_ESM.zip › Supplementary Figure 1R2.tif]

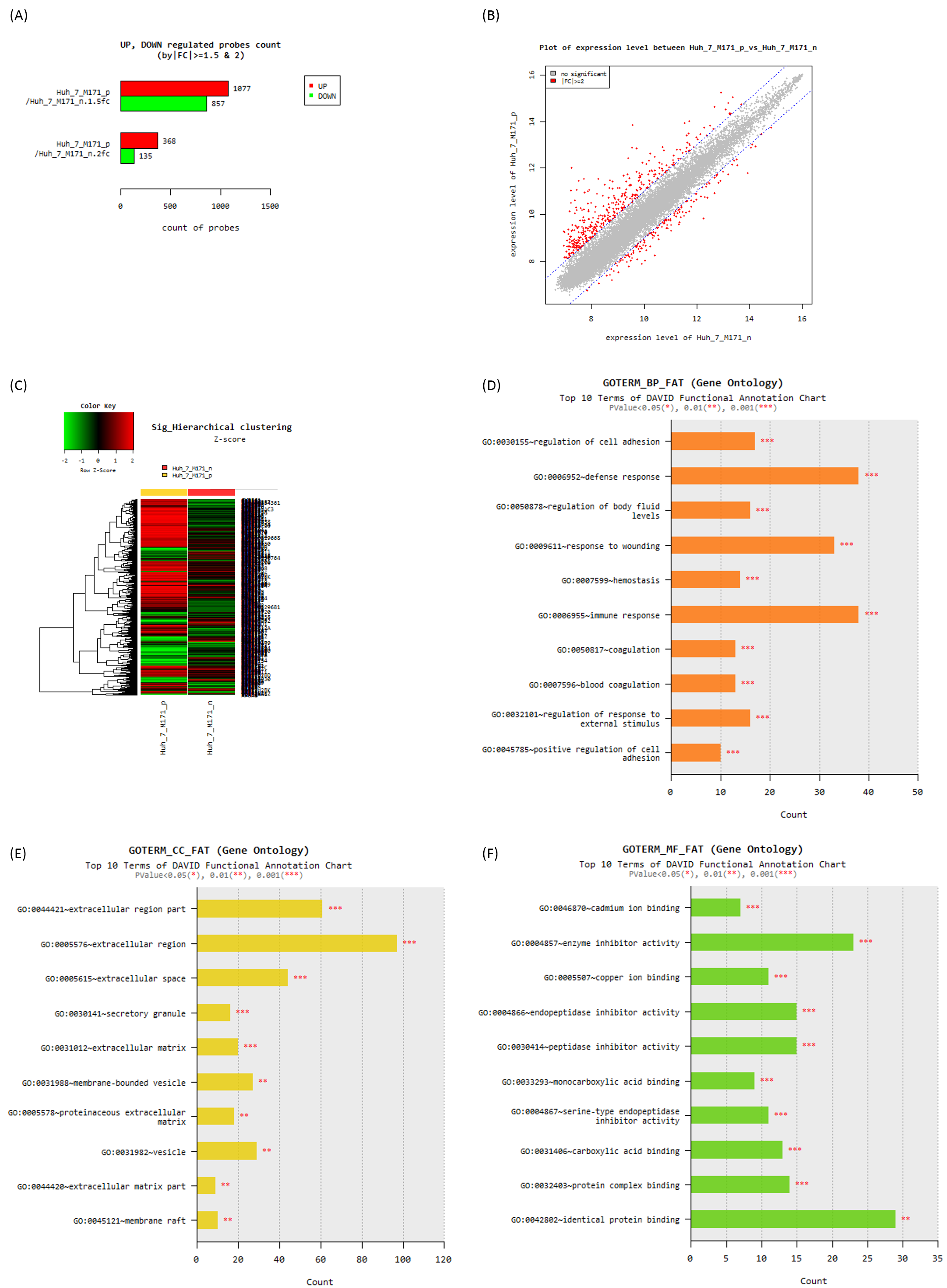

Supplement: Supplementary file 1 — Additional file 1: Table S1. A list of genes up-regulated with fold change ≥2 in cDNA microarray analysis. Table S2. Growth inhibition rates of each treatment. Figure S1. The cytotoxic efficacy of CKD-5 and panobinostat in SNU-761 cells. Both panobinostat and CKD-5 treatment reduced cell proliferation, and the cytotoxic efficacy was more potent in CKD-5 than panobinostat especially when combined with Sorafenib. Figure S2. Results of cDNA microarray assay. (A) The number of up- or down-regulated probes filtered by P-value and various fold changes. (B) A scatter plot of expression level between the control and HDACi-treated samples. (C) Hierarchical clustering heatmap. (D-F) Gene-enrichment and functional annotation analysis using DAVID based on Gene Ontology database. Figure S3. Changes in tumor volume over time after each treatment in a model of C3H mouse implanted with MH-134 cells. Combination therapy of high dose CKD-5 with sorafenib significantly suppressed tumor growth more than any other treatment. Figure S4. The degree of apoptosis in (A) liver and (B) spleen tissue assessed by TUNEL and H&E staining. Little apoptosis of the liver and spleen tissue was detected in all treatment groups. [file 12885_2020_7471_MOESM1_ESM.zip › Supplementary Figure 2R2.tif]

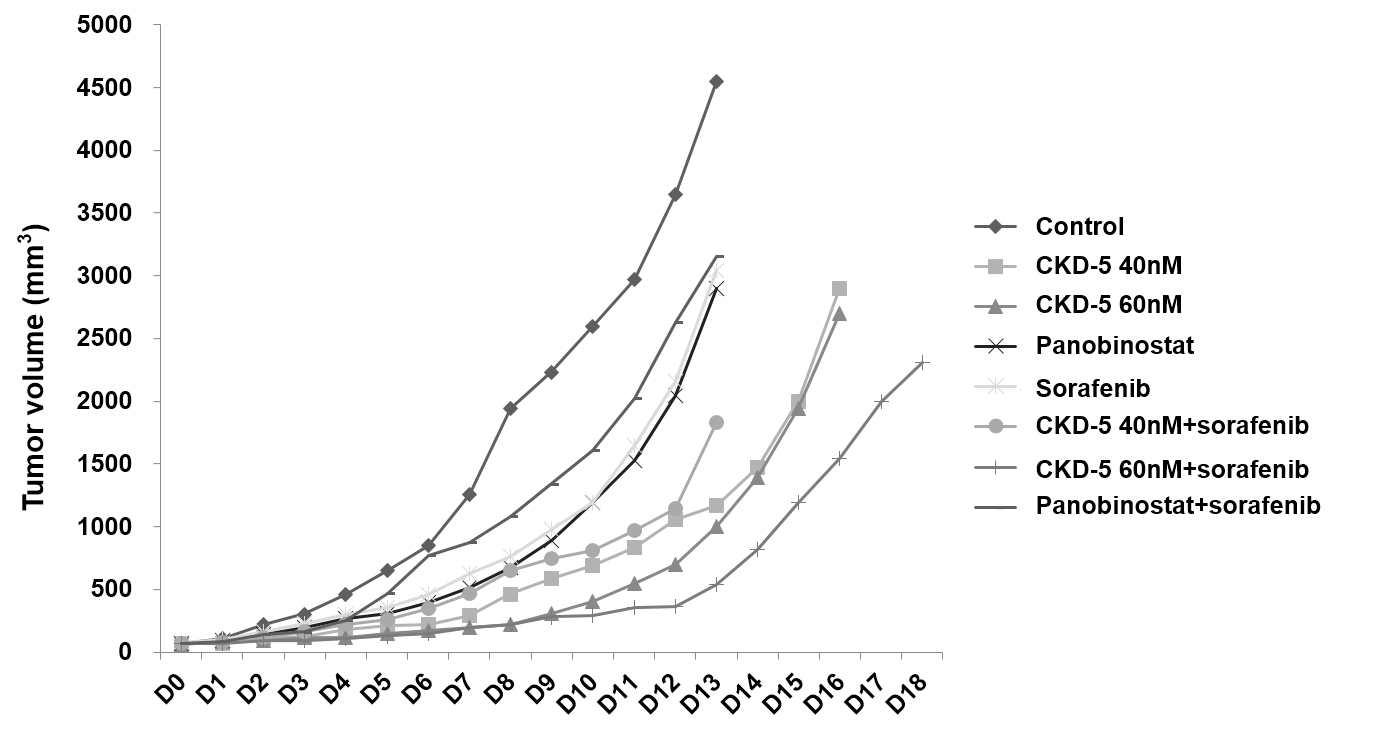

Supplement: Supplementary file 1 — Additional file 1: Table S1. A list of genes up-regulated with fold change ≥2 in cDNA microarray analysis. Table S2. Growth inhibition rates of each treatment. Figure S1. The cytotoxic efficacy of CKD-5 and panobinostat in SNU-761 cells. Both panobinostat and CKD-5 treatment reduced cell proliferation, and the cytotoxic efficacy was more potent in CKD-5 than panobinostat especially when combined with Sorafenib. Figure S2. Results of cDNA microarray assay. (A) The number of up- or down-regulated probes filtered by P-value and various fold changes. (B) A scatter plot of expression level between the control and HDACi-treated samples. (C) Hierarchical clustering heatmap. (D-F) Gene-enrichment and functional annotation analysis using DAVID based on Gene Ontology database. Figure S3. Changes in tumor volume over time after each treatment in a model of C3H mouse implanted with MH-134 cells. Combination therapy of high dose CKD-5 with sorafenib significantly suppressed tumor growth more than any other treatment. Figure S4. The degree of apoptosis in (A) liver and (B) spleen tissue assessed by TUNEL and H&E staining. Little apoptosis of the liver and spleen tissue was detected in all treatment groups. [file 12885_2020_7471_MOESM1_ESM.zip › Supplementary Figure 3R2.tif]

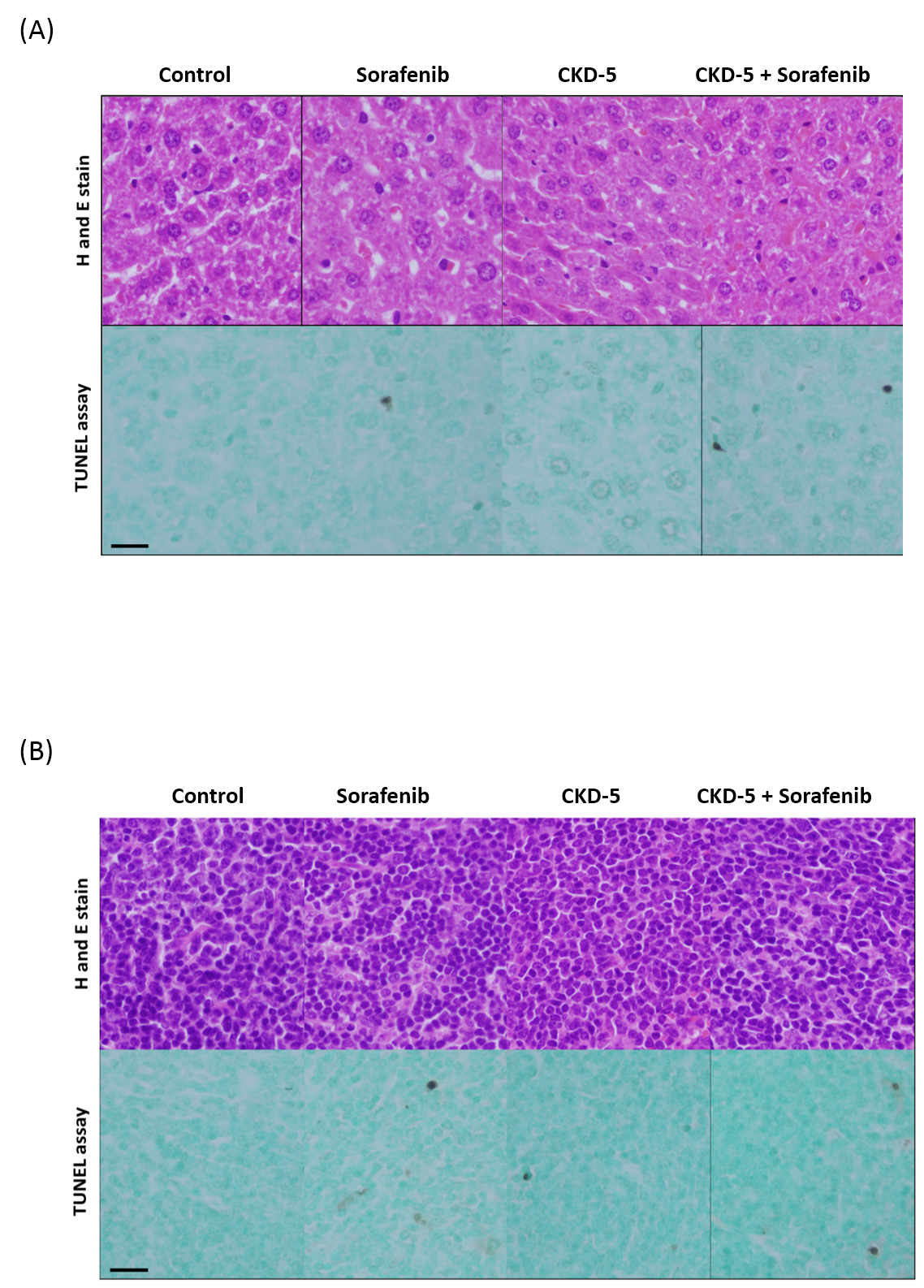

Supplement: Supplementary file 1 — Additional file 1: Table S1. A list of genes up-regulated with fold change ≥2 in cDNA microarray analysis. Table S2. Growth inhibition rates of each treatment. Figure S1. The cytotoxic efficacy of CKD-5 and panobinostat in SNU-761 cells. Both panobinostat and CKD-5 treatment reduced cell proliferation, and the cytotoxic efficacy was more potent in CKD-5 than panobinostat especially when combined with Sorafenib. Figure S2. Results of cDNA microarray assay. (A) The number of up- or down-regulated probes filtered by P-value and various fold changes. (B) A scatter plot of expression level between the control and HDACi-treated samples. (C) Hierarchical clustering heatmap. (D-F) Gene-enrichment and functional annotation analysis using DAVID based on Gene Ontology database. Figure S3. Changes in tumor volume over time after each treatment in a model of C3H mouse implanted with MH-134 cells. Combination therapy of high dose CKD-5 with sorafenib significantly suppressed tumor growth more than any other treatment. Figure S4. The degree of apoptosis in (A) liver and (B) spleen tissue assessed by TUNEL and H&E staining. Little apoptosis of the liver and spleen tissue was detected in all treatment groups. [file 12885_2020_7471_MOESM1_ESM.zip › Supplementary Figure 4R2.tif]
